# Evidence of Cognitive Dysfunction after Soccer Playing with Ball Heading Using a Novel Tablet-Based Approach

**DOI:** 10.1371/journal.pone.0057364

**Published:** 2013-02-27

**Authors:** Marsha R. Zhang, Stuart D. Red, Angela H. Lin, Saumil S. Patel, Anne B. Sereno

**Affiliations:** 1 University of Texas Health Science Center at Houston, Houston, Texas, United States of America; 2 Baylor College of Medicine, Houston, Texas, United States of America; University of Milan, Italy

## Abstract

Does frequent head-to-ball contact cause cognitive dysfunctions and brain injury to soccer players? An iPad-based experiment was designed to examine the impact of ball-heading among high school female soccer players. We examined both direct, stimulus-driven, or reflexive point responses (Pro-Point) as well as indirect, goal-driven, or voluntary point responses (Anti-Point), thought to require cognitive functions in the frontal lobe. The results show that soccer players were significantly slower than controls in the Anti-Point task but displayed no difference in Pro-Point latencies, indicating a disruption specific to voluntary responses. These findings suggest that even subconcussive blows in soccer can result in cognitive function changes that are consistent with mild traumatic brain injury of the frontal lobes. There is great clinical and practical potential of a tablet-based application for quick detection and monitoring of cognitive dysfunction.

## Introduction

Concussive brain injuries in head-jarring sports such as American football, hockey, and boxing, where repeated loss of consciousness often occur, could lead to long-term cognitive dysfunctions [1]. However, whether less violent head impacts such as heading a soccer ball could lead to subconcussive brain injury is unclear [2]–[4]. A recent imaging study [5] showed detectable structural differences in brain areas, consistent with traumatic brain injury (TBI), between amateur adult (mean age of 31 yrs, played soccer since childhood) soccer players with self-reported high and low heading frequencies. Similar findings were also obtained in another recent imaging study [6] which found differences in white matter integrity in a small sample of professional male soccer players (mean age of 20 yrs, who played soccer since childhood) compared with a control group of swimmers (mean age of 21 yrs). Previous imaging studies have failed to find structural brain differences directly related to heading balls [7]–[10]. Previous studies using formal cognitive testing have also failed to detect changes with ball heading in young adults [11] or in 13- to 16-year-old soccer players

Previous studies that did not find significant changes in higher level cognitive tasks associated with soccer ball heading [10], [11] have often tested for cognitive changes using more formal but complicated cognitive testing (e.g., visual memory retention, addition, logic, and other tasks that occur at the level of seconds and minutes). Moreover, several studies have now demonstrated that standard neuropsychological testing is less sensitive than eye tracking tasks in detecting differences in cognitive or executive function [12]–[14]. For example, [12]showed that eye tracking (or oculomotor) biomarkers were more sensitive to treatment-related changes in neurocognitive function than traditional neuropsychological measures [12]. Here we use a new touch based method, with tasks similar to those used in eye tracking research, which are simple, straightforward, and less sensitive to interfering issues such as second language differences. Our method with its relatively short response latencies and high temporal resolution may be a more sensitive test of executive function and hence be able to dectect more subtle cognitive changes in high school soccer players, deficits that were previously undetected because of lack of sensitive measurement techniques.

Frontal lobes are among the brain regions most susceptible to injury in traumatic brain injury [15]. Using a variant on a well-established frontal-lobe task of executive function (antisaccade task), we developed a simple iPad-based application to detect the cognitive effects of soccer ball heading. Prior research shows that the antisaccade task, a voluntary eye movement task, can be used to determine deficits in executive functioning [16]–[19]. Point responses by the hand towards a target (Pro-Point) are similar to prosaccade eye movements in that they both involve a more reflexive motor movement directly to a target; while point responses away from a target (Anti-Point) are more similar to antisaccade movements as they involve inhibition of the reflexive movement towards the target and generation of a voluntary or goal directed motor movement in the opposite direction of the target location [20]–[22]. We hypothesize that ball heading causes cognitive dysfunctions as measured by soccer player’s slower responses and more errors in Anti-Point tasks, indicating disruption of their voluntary brain function.

## Methods

The participants were 12 female soccer and 12 female non-soccer players in a high school (median age for both groups  =  16.5 years; range for both groups was 15–18 years). Both soccer and non-soccer players were recruited through the high school, and a research assistant explained the study to them. All participants gave informed consent or assent with parental consent and the study was approved by the University of Texas at Houston Committee for the Protection of Human Subjects in accordance with the Declaration of Helsinki. In addition, the study was also approved by the administration of the high school as well as the coaches. Every soccer player performed head balls during the practice session before the testing, with median 6 (range: 2–20) head balls per session based on self-reports. Data for two of the soccer subjects were not included in the descriptive statistics of heading ball rate or used for the analysis of this variable as their answers were qualitative. No participant in the non-soccer group performed a head ball before testing. The medians (and ranges) for years of soccer playing and current weekly hours of soccer playing were respectively 8 years (range: 5–12) and 11 hours (range: 2–16), for soccer players and 0 and 0 for non-soccer players. The non-soccer players were recruited similar to soccer players with the additional inclusion criteria that they were not currently active in a contact sport and that their age and grade level was matched to the soccer players. All participants had normal or corrected-to-normal vision and none reported any previous head injury nor any other known neurological conditions. The medians for the numbers of hours of video (electronic) game playing were 4.0 and 2.5 hours for soccer and non-soccer players. Eleven of the twelve participants in both the soccer and non-soccer groups were right handed.

### Stimuli

**Figure 1:**
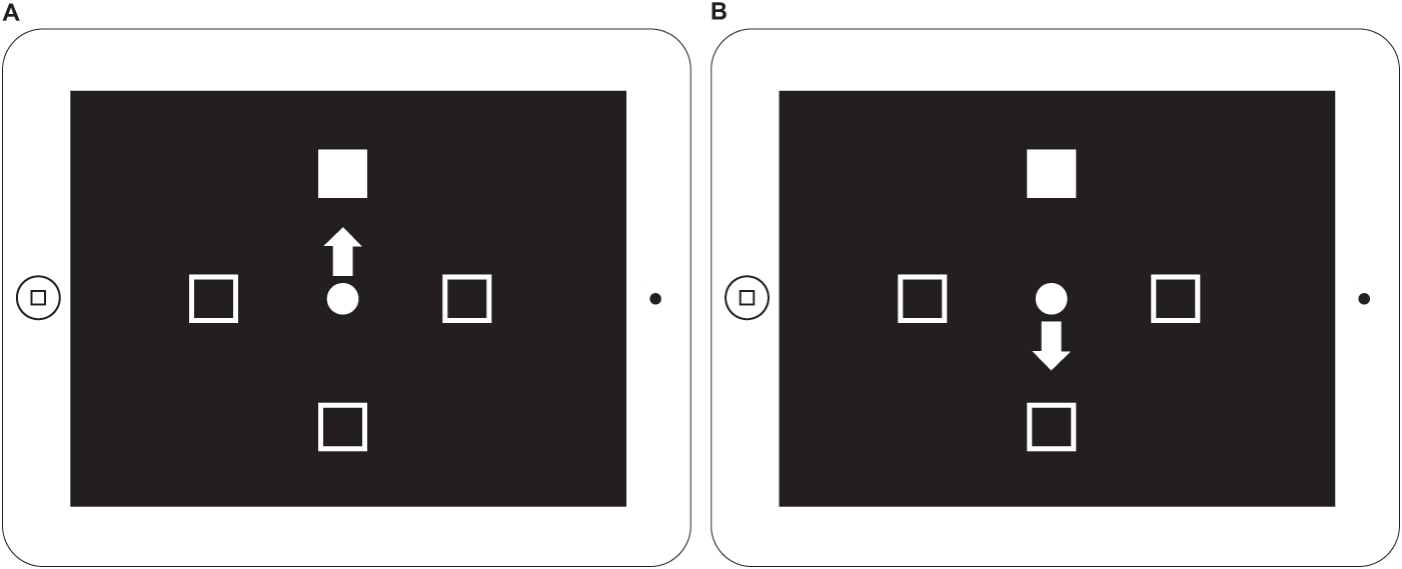
Schematic of the two tasks displayed on iPad screen. **A.** Pro-Point task on the iPad with white arrow indicating direction of a correct response (toward the target square). **B.** Anti-Point task on the iPad with white arrow indicating direction of a correct response (away from the target square).

The experiment was performed on an iPad 2 (Figure 1) with a video frame refresh rate of 60 Hz. The onset and offset of stimuli were synchronized with the frame refresh signal with a precision of 1.6 msec [23]. The visual display consisted of a filled center fixation circle (diameter subtending 2.4° visual angle from a 33 cm viewing distance, 1.4 cm) surrounded by four square boxes (1.4°, 0.8 cm) 7.0° (4.0 cm) from the fixation circle indicating possible response locations. Participants started a trial by placing their index finger on the center circle. A visual target (white square, 0.8 cm) appeared randomly 480 ms later, at one of the four locations. For the Pro-Point task, the participant was instructed to touch the response box containing the target as fast as possible without making errors. For the Anti-Point task, the participant was to touch the response box opposite to the target location.

### Touch responses

The spatial locations of the touches were captured by the iPad’s capacitive touch screen and exact coordinates calculated using its touch-screen interface with resolution of 52 pixels per cm. A response was counted as an error if the distance between the target location and the iPad-calculated location was greater than 3.3° (1.9 cm). The touch screen alone cannot be used for high temporal resolution measurements because of the inherent delay associated with sensing touch via a capacitive screen as well as the fact that these events are then discretized to the frame refresh rate of 60 Hz. A temporal resolution of 0.2 msec was achieved by using the built-in microphone (44.1 kHz sampling rate) on the iPad to record the vibrations produced by touch onset and offset [23].

### Design and Procedure

The dependent variables were: (1) Initiation Time - the duration between when the visual cue appears and when the finger is lifted, (2) Movement Time - the duration between when the finger is lifted and when the target goal (at the cue or opposite of the cue location) is touched, (3) Total Time - the duration between when the visual cue appears and when the target goal is touched, and (4) Error - when the finger touched more than 3.3° (1.9 cm) from the target goal center. Each participant performed two blocks of trials until they obtained 48 correct trials in each Pro-Point and Anti-Point block (mean total trials 48.3 and 49.4, respectively). Within each participant group, half started with the Pro-Point block followed by the Anti-Point block, and the other half received the reversed order. Both groups were tested after school academic activities were over. Soccer players went to practice right after school academic activities and then were tested in the field immediately following their afternoon practice. To match the environmental conditions, Non-soccer players were also tested outdoors after school academic activities were over at approximately the same time in the afternoon (4–5 pm).

### Analysis

Error trials were excluded from the analyses of response times. Outlier trials with times more than 2.5 SD away from the mean of each subject for each task were excluded iteratively until all remaining trails were within 2.5 SD, removing, for initiation times, 6.86% (Pro-Point) and 3.91% (Anti-Point) and, for movement times, an additional 0.95% and 2.60% of the total trials, respectively. A mixed effect model was performed for response time data and a logit-link generalized linear model with repeated measurements for error data. The logit-link transforms error percentage, p, to logit(p) by log[p/(1-p)]. All models assumed that measurements obtained within each subject have an autoregressive correlation structure, AR(1). Group (soccer vs. non-soccer players) was the between-subject variable for each task (Pro-Point and Anti-Point) and group difference was calculated and tested by constructing the contrasts from the mixed effect models or logit-link generalized linear model. In addition, to test if the Anti-Point response time slowing in the soccer group found in the first group analysis was related to ball heading, years of soccer, or current weekly hours of soccer playing, we performed a similar mixed effect model (with repeated measurements and autoregressive correlation structure) on the Anti-Point response time data from the soccer players with the independent variables of heading rate (n = 10), years of soccer playing (n = 12), and hours of soccer per week (n = 12). Due to missing data, analyses were run separately. Data were analyzed using a significance level of p<0.05 and a marginal significance level of. 05≤p<0.10.

## Results

**Figure 2:**
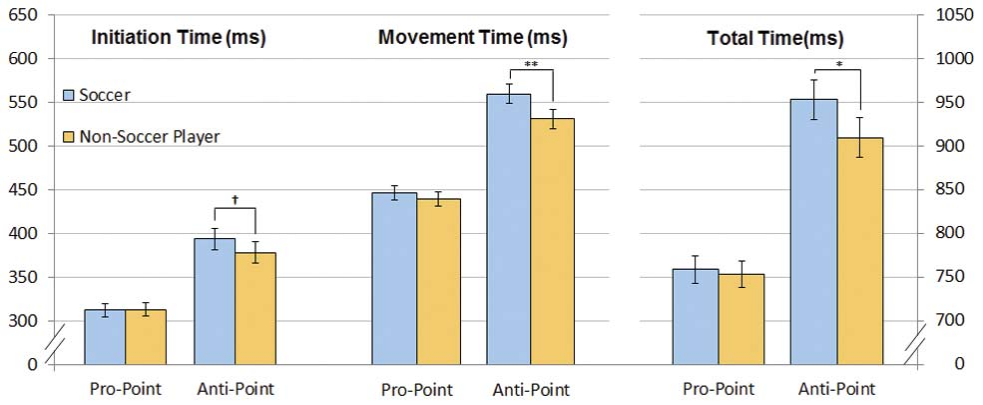
Estimated mean initiation, movement, and total reaction times for soccer and non-soccer subjects. Blue bars represent data from soccer players, and orange bars represent data from non-soccer players. Error bars indicate the 95% confidence interval (d.f. = 11). Note that the scale for total time (far right) is different from that of the initiation and movement times. Significance levels: (†) for p<0.1, (*) for p≤0.01, and (**) for p<0.005.

Figure 2 illustrates mean initiation, movement, and total times to respond to the target as a function of task for both groups. In Pro-Point, there were no differences between soccer and non-soccer players for initiation times (312 ms vs. 313 ms, t(22) = 0.16, p = 0.87), movement times (445 ms vs. 439 ms, t(22) = 1.18, p = 0.25), or total times (757 ms vs. 752 ms, t(22) = 0.55, p = 0.59) using the mixed effect model. In contrast, in Anti-Point, soccer players were marginally slower than non-soccer players for initiation times (394 ms vs. 378 ms, t(22) = 1.86, p = 0.08) and significantly slower than non-soccer players for movement times (561 ms vs. 531 ms, t(22) = 3.69, p<0.005) and total times (955 ms vs. 909 ms, t(22) = 2.81, p = 0.01) using the mixed effect model.

To further test if Anti-Point response times in soccer players could be accounted for by heading frequency or soccer experience, we included these three variables as independent variables. The mixed effect model with heading ball rate as the independent variable showed marginal effects of heading ball rate for initiation time (t(8) = 1.88, p<0.10) and total time (t(8) = 1.86, p<0.10), but not movement time (t(8) = 1.67, p>0.11) in the Anti-Point task, indicating marginally slower responses with increased number of head balls. With hours of soccer per week as the independent variable, the mixed effect model showed signicant effects for Anti-Point task initiation time (t(10) = 3.51,p<.01), movement time (t(10) = 2.27, p<.05) and total time (t(10) = 2.95, p<02), indicating slower responses with increased hours of soccer per week. Thirdly, with years of soccer as the independent variable, the mixed effect model showed a marginal effect for Anti-Point task initiation time (t(10) = 2.94,p<.07) and signifcant effects for movement time (t(10) = 3.43, p<01) as well as total time (t(10) = 2.71, p<03), indicating slower responses with increased years of soccer experience. Finally, it was determined that the independent variables of heading ball rate, hours of soccer played per week, and years of soccer played were unrelated/uncorrelated by Pearson correlation coefficients.

There were no significant differences in errors in either Pro-Point tasks (0.3% vs. 1.0%, z = 0.83, p = 0.41, logit-link generalized model) or Anti-Point (3.1% vs. 2.5%, z = 1.13, p = 0.26, for soccer and non-soccer players, logit-link generalized model).

### Conclusion

The results show that soccer playing in which participants headed the ball did indeed disrupt voluntary performance in female high school soccer players tested immediately following practice. In addition, even in this small sample, this response time slowing on the Anti-Point task was marginally related to number of ball headers (n = 10) and significantly related to hours of soccer per week (n = 12) and years of soccer playing (n = 12). We found no evidence that slowing occurred during reflexive movements under identical sensorimotor conditions (Pro-Point). These findings demonstrate significant and specific cognitive changes in female high school soccer players who head the soccer ball during practice.

One alternative explanation for finding RT slowing after soccer playing is that information processing might be slowed immediately following a bout of aerobic exercise. However, we found no evidence of slowing for the Pro-Point task with identical sensory and motor demands in soccer players. Further, studies that have specifically examined the effects of exercise on cognitive performance have shown *reduced* RTs and *enhanced* cognitive functioning and memory storage and retrieval following acute aerobic exercise [24], [25]. Thus, the disruption of cognitive performance in soccer players in our study was not likely due to aerobic activities immediately preceding the testing session. Moreover, such studies, showing enhanced cognitive performance following aerobic exercise, suggest instead that the differences we report might underestimate the cognitive slowing that occurs after soccer playing with ball heading. Nevertheless, given our findings, future work should explore in soccer players the effect of aerobic exercise on cognitive performance independently of ball heading.

Some previous studies in more advanced soccer players have demonstrated correlations in cognitive disruptions with frequency of lifetime heading [26], [27]; however recent studies using computerized testing and more appropriate groups have rebutted these claims and suggested that lifetime heading is not correlated with cognitive deficits [2], [28]. Further, previous studies in young players have also failed to detect any cognitive changes [28], [29]. We found evidence in our small sample of young soccer players that soccer playing with ball heading did result in cognitive changes and that these increases in response time on the Anti-Point task related to both number of headers, hours of soccer per week, and duration of soccer experience. The most conservative interpretation of our findings is that these changes are transient and the result of the immediately preceding soccer session. The trend for an increase in response time with number of ball headers would support such an interpretation. To be consistent with this interpretation, it is possible that the response time slowing that relates to hours of soccer played per week and years of experience is only measurable immediately following these additional subconcussive blows (conceptually similar to the phenomenon of drug sensitization, where there is amplification of a response due to prior experience). Alternatively, the significant slowing that relates to hours of soccer played per week and years of experience could be interpreted to indicate longer lasting changes cumulative across days, the sports’ season, and years of experience. We are unable in our study to tease apart immediate transient effects from longer lasting effects due to the study design. Without additional data, we prefer a more conservative interpretation of transient deficits due to the immediately preceding soccer practice with ball heading. It is also possible that there exists a relationship between the independent variables of heading ball rate, years of soccer played, and hours of soccer played per week that was unattainable with our small sample. Larger scale follow up studies may show that these variables are working together to drive these effects rather than working separately as we have shown here.

The study was carried out at a local high school that was supportive of the study but wanted to minimize any inconvenience to the students, their parents (for minors under 18), and their sports schedule. The soccer sessions were actual varsity training sessions that were on their own tight schedule and it was not possible to run a pre-practice control. The varsity coach controlled the practice, including the heading portion, and we did not have control over what soccer related activity the players performed. There are many potential follow up studies that are possible to try to control for specific differences between soccer and non-soccer players that are difficult to control in a more observational rather than randomized design. Observational designs, where the assignment of treatments is beyond the control of the investigator, can be chosen for a number of reasons, including lack of influence (e.g., on structuring varsity practices) as well as concerns about violation of ethical standards. On the other hand, observational designs provide an accurate assessment of real-world use and practice and help to formulate hypotheses to be tested in following experiments (such as whether the effect we report is transient, and reduced or absent by the next day, accumulative across the week or season, or dependent on a previous history of subconcussive blows).

In sum, the cognitive changes that we report were measured with a simple iPad based application. A simple tool such as this iPad application may be a quick and effective method to screen for and track cognitive deficits in sports players. It could potentially be used to detect, screen, and track other populations for mild traumatic brain injury and development of cognitive comorbidities.

Though the changes we report were robust, they do not necessarily imply sustained changes or brain injury. Further study is needed to track soccer players for longer periods to evaluate if these changes are transient or longer-lasting, if they are dependent upon repeated subconcussive blows, and if they generalize to male soccer players. To our knowledge, these results provide the first evidence that even subconcussive blows in soccer could lead to measureable, even if possibly transient, cognitive changes in young soccer players.
