# Correction: Evidence of Cognitive Dysfunction after Soccer Playing with Ball Heading Using a Novel Tablet-Based Approach

**DOI:** 10.1371/journal.pone.0200450

**Published:** 2018-07-05

**Authors:** Marsha R. Zhang, Stuart D. Red, Angela H. Lin, Saumil S. Patel, Anne B. Sereno

There is an error in the first sentence of the “Design and Procedure” subsection of the Methods. The correct sentence is: The dependent variables were: (1) Initiation Time—the duration between when the visual cue appears and when the finger is lifted, (2) Movement Time—the duration between when the visual cue appears and when the target goal (at the cue or opposite of the cue location) is touched, (3) Total Time—the sum of Initiation Time and Movement Time, and (4) Error—when the finger touched more than 3.3° (1.9 cm) from the target goal center.

With these changes, the dependent variable Initiation Time (which is defined correctly in the original article) is similar to the dependent variable often measured in saccade paradigms called saccade latency, and the dependent variable Movement Time (which is defined incorrectly in the original article, and is corrected in the paragraph above) is similar to the dependent variable often measured in manual response tasks called response time. Finally, Total Time is the sum of these two dependent variables. This correction in the description of the dependent variables is important so that they are accurately defined and can be properly related to the previous literature. Note that the constructed variable Total Time is a linear combination of the other two variables, which double weights the time from onset of the cue until finger lift and single weights the time from finger lift until target goal is touched. The timing data presented in Figure 2 of the published article are correct and reflect the new definitions provided here.

The authors confirm that these changes do not change any of the statistical findings, interpretations, or conclusions reported.

## References

[1] Zhang MR, Red SD, Lin AH, Patel SS, Sereno AB (2013) Evidence of Cognitive Dysfunction after Soccer Playing with Ball Heading Using a Novel Tablet-Based Approach. PLoS ONE 8(2): e57364 https://doi.org/10.1371/journal.pone.0057364 23460843 10.1371/journal.pone.0057364 PMC3583826

